# Investigating significant health trends in progressive fibrosing interstitial lung disease (INSIGHTS-ILD): rationale, aims and design of a nationwide prospective registry

**DOI:** 10.1186/s12890-023-02333-7

**Published:** 2023-02-11

**Authors:** Juergen Behr, Francesco Bonella, Andreas Günther, Dirk Koschel, Antje Prasse, David Pittrow, Jens Klotsche, Michael Kreuter, INSIGHTS-ILD Study Group, Ioana Andreica, Jürgen Behr, Heike Biller, Martin Claussen, Stephan Budweiser, Stephan Eisenmann, Ralf Ewert, Wolfgang Gesierich, Sven Gläser, Christian Grohé, Daniel Grund, Achim Grünewaldt, Lars Hagmeyer, Matthias Held, Hans-Joachim Kabitz, Joachim Kirschner, Philipp Markart, Ulrich Neff, Claus Neurohr, Frank Reichenberger, Peter Schramm, Martin Schwaiblmair, Bernd Seese, Dirk Skowasch, Julia Wälscher, Michael Weber, Michael Westhoff, Heinrike Wilkens, Hubert Wirtz

**Affiliations:** 1 Department of Medicine V, Comprehensive Pneumology Center, Member of the German Center for Lung Research (DZL), University Hospital, LMU Munich, Munich, Germany; 2 Center for Interstitial and Rare Lung Diseases, Pneumology Department, Ruhrlandklinik University Hospital, Duisburg-Essen University, Essen, Germany; 3 Universities of Giessen and Marburg Lung Center (UGMLC), Member of the German Center for Lung Research (DZL), Giessen, Germany; 4 Medical Department I, Division of Pneumology, University Hospital Carl Gustav Carus, and Department of Pulmonology, Fachkrankenhaus Coswig GmbH, Lung Center, Coswig, Germany; 5 Department of Respiratory Medicine, Hannover Medical School, Hannover, Germany; 6 Institute for Clinical Pharmacology, Medical Faculty, Technical University, Dresden, Germany; 7 German Rheuma Research Center (DRFZ), Epidemiology, Berlin, Germany; 8 Center for Interstitial and Rare Lung Diseases, and Interdisciplinary Center for Sarcoidosis, Pneumology, Thoraxklinik, University Hospital Heidelberg, German Center for Lung Research, Heidelberg, Germany; 9 Department of Pneumology, RKH Clinics Ludwigsburg, Ludwigsburg, Germany

**Keywords:** Interstitial lung disease, Lung fibrosis, Observational trial, Longitudinal, Health care, Outcomes, Ambulatory setting, Quality of life

## Abstract

**Background:**

The progressive course of pulmonary fibrosis (PPF) is observed with variable prevalence in different entities of fibrosing interstitial lung disease (fILD). PPF is characterised by worsening respiratory symptoms, declining lung function and increasing extent of fibrosis on high-resolution computer tomography. In Germany, data are limited on the characteristics and management of such patients.

**Methods/Design:**

INSIGHTS-ILD is a prospective observational longitudinal registry designed to describe characteristics, management and course of newly diagnosed (incident) and prevalent patients with fILD on the long term. The registry uses a non-probability sampling approach to collect data on characteristics, therapeutic interventions, health-related quality of life and health economic parameters. It is planned to include 900 patients in ambulatory care in about 30 expert sites over three years. The study has been initiated in December 2021, and currently (January 2023) follows 360 patients.

**Discussion:**

The registry is expected to provide much-needed data on the characteristics, management, and trajectories of patients fILD in Germany. The start of the study comes at a time when new treatment options are available for PPF. We hypothesize that PPF represents a broad clinical phenotype that is differentially influenced by inflammatory and fibrotic pathomechanisms that need to be treated with anti-inflammatory and/or anti-fibrotic treatment strategies. This registry will allow comparisons with other countries. Gap analyses based on current guidelines for management of these patients will be possible.

*Trial registration* DRKS00027389 (registered on 7.12.2021), BfArM NIS 7562.

## Background

The term interstitial lung disease (ILD) describes a large, heterogeneous group of more than 200 disorders affecting the lung parenchyma with overlapping clinical, radiographic, and histopathologic manifestations [1]. ILDs have differing underlying aetiologies, treatment options, clinical presentations and courses [2]. The most common and most studied form of fibrosing ILD is idiopathic pulmonary fibrosis (IPF).

Overall, early and accurate (differential) diagnosis of ILD can be challenging, and it is difficult to predict disease progression and treatment response in an individual patient [3].

The progressive course of non-IPF pulmonary fibrosis has been termed PPF in the most recent international guideline [4]. PPF covers large sub-groups of patients with fibrotic lung diseases that clinically progress despite appropriate usual management and is now an everyday problem for patients and clinicians alike [5]. Pulmonary fibrosis is typically regarded as an indicator and predictor of disease progression, and the prognosis associated with an ILD is generally worsened by the development and/or progression of fibrosis [6].

Progressive fibrosis is characterized by worsening respiratory symptoms, deterioration in lung function, limited response to immunomodulatory therapies, decreased quality of life and, potentially, early death. However, aside from IPF, fibrosing ILDs may be clinically stable, especially if the disease is mild. To date, several criteria for “progressive fibrosis” have been defined. It has been suggested that patients meeting any of the following criteria within a 24-month period have experienced disease progression: a relative decline of ⩾ 10% in forced vital capacity (FVC); a relative decline of ⩾ 15% in diffusing capacity of the lung for carbon monoxide (DLCO); or worsening symptoms or a worsening radiological appearance accompanied by a ⩾5– < 10% relative decrease in FVC [3, 6, 7]. In the most recent ATS/ERS/JRS/ALAT practice Guideline the guideline committee, suggested the presence of two out of three categories: 1. worsening respiratory symptoms, 2. worsening lung function or 3. increase of fibrosis on HRCT morphology within one year to make a diagnosis of PPF. It is commonly accepted that these criteria are only valid if other reasons for worsening have been excluded [4].

Improved survival from this disease spectrum may be dependent on better understanding of the epidemiology of the disease, its diagnostic spectrum and an analysis of outcomes from emerging therapies at a significant level [8]. Therefore, it is important to obtain detailed data on the natural course of fibrosing ILDs and their PPF phenotype, to understand patient characteristics, the situation and management of these patients in clinical practice.

Among the PPF, in Germany the collection of real-world data has focused on IPF (e.g. eurIPFnet [9], INSIGHTS-IPF [10–12]), and to a lesser extent on the other ILDs (GOLDnet [13], EXCITING [14]).

The newly initiated registry INSIGHTS-ILD can document fibrosing ILDs and their PPF in the context of the introduction of new therapies and complement the data from randomised controlled trials. It may serve quality assurance purposes, as individual centres can compare their results with other centres and with what is stated in guidelines.

## Rationale and aim

The present INSIGHTS-ILD disease registry aims to provide comprehensive information on different aspects of PPF within routine clinical practice: patient characteristics, diagnosis, management (including utilization, effectiveness and safety of medication), clinical course, quality of life and other patient related outcomes (PRO), and economic variables.

It documents all PPF with the exception of IPF since the latter entity has been documented in great detail in another registry named INSIGHTS-IPF [10–12].

## Objectives and endpoints

The objectives and corresponding endpoints of the registry are shown in Table 1.

**Table 1:** Objectives and endpoints

	Objectives	Endpoints
Primary	To compare various PPF groups and treatment strategies in terms of characteristics and outcomes (lung function, survival, quality of life, other)	Time to progression of disease (any one of the following): Decrease of FVC > 10% predicted within one year Decrease Dlco > 15% predicted plus initiation of LTOT or permanent increase of oxygen flow when LTOT is established within one year Decrease of 6-MWD > 50 m within one year Hospitalization due to respiratory decompensation Death of any cause Change of treatment strategy (stop of anti-inflammatory or antifibrotic therapy, initiation of new anti-inflammatory or antifibrotic therapy or a combination of both)
Secondary	To assess drug utilization	Treatment strategy (immunosuppression, antifibrotic therapy) Dose and dosing schedule Duration of treatment (persistence) Switches between treatments Phases without drug treatment (with reasons) Reasons for drug discontinuation
	1. To determine risk factors for progression of disease 2. To determine factors for treatment success or failure, respectively, defined as clinical, functional, and radiographic stabilization (i.e. absence of deterioration in each of the categories) and change of treatment strategy (i.e. stop/start of antifibrotic therapy; stop/start of immunosuppressive therapy)	Survival Clinical symptoms Dyspnea Cough Lung function Annual DLCO decline Annual FVC decline 6-min walk distance QoL scores over time Radiographic course: fibrosis on HRCT Therapy escalation Clinical events (exacerbations, hospitalisations) Analyses stratified according to different diagnosis groups
	3. To determine potential biomarkers, which are routinely measured in many centers, to differentiate inflammatory driven from fibrosis driven progress	Laboratory parameters: CRP LDH Differential blood cell count (lymphocytes, neutrophils, eosinophils, monocytes), BAL differential cell count (as available)
	To assess safety	Adverse Events Adverse Drug Reactions

## Ethical aspects and data protection

### Ethical approval

The registry is conducted consistent with consensus ethics principles derived from international ethics guidelines including the Declaration of Helsinki, and applicable local regulatory requirements and laws.

Prior to any data collection under this protocol, written informed consent with the approved ICF is obtained from the patient or patient’s legally authorized representative, as applicable, in accordance with local practice and regulations.

Detailed information about the registry and that registry participation is voluntary will be explained to the patient. This information may be provided in a personal talk, and alternatively via telephone or videoconference. The patient will be given sufficient time to consider whether to participate in the registry. The signed ICF will be retained with the registry records. A copy of the ICF, signed and dated by the patient, must be given to the patient.

The patient can revoke registry participation at any time. As long as a patient does not recant the use of personal data, these data will be included in the intended analyses if applicable, even if the patient has resigned from the registry. Patients who have revoked their participation will not be replaced.

### Confidentiality and data protection

The sponsor as well as all investigators ensure adherence to applicable data privacy protection regulation. Data are transferred in encoded form only. The entire documentation made available to the sponsor does not contain any data which, on its own account or in conjunction with other freely available data, can be used to re-identify natural persons. The investigators are obligated to ensure that no documents contain such data.

All records identifying the subject will be kept confidential and will not be made publicly available. Patient names will not be supplied to the sponsor. If the patient’s name appears on any document, it must be obliterated before a copy of the document is supplied to the sponsor. Registry findings stored on a computer will be stored in accordance with local data protection laws.

The investigator will maintain a list to enable patients’ records to be identified in case of queries. In case of a report of a serious adverse drug reaction (SADR), the responsible pharmacovigilance person may ask for additional clarification. In that case, the company is not allowed to directly contact the patient. All additional information will be provided by the investigator.

Data protection rules will be closely followed and the standards of the General Data Protection Regulation (GDPR) will be met. Neither initials nor the exact birth date of the patient will be recorded in the database. Patient data are collected under a pseudonym. Upon saving of the first assessment (= inclusion visit), the EDC system automatically assigns a unique and consecutive Subject Identification Code (SIC). All registry documents (e.g., print outs of electronic case report forms, the informed consent forms etc. are identified with the SIC. The SIC alone cannot be used for identification of a given patient outside a site, in compliance with laws governing data privacy.

## Observational plan

### Design

This ILD registry allows for structured, non-interventional collection of data. Participating physicians will not be subject to any instructions with regard to the diagnosis and therapy of their patients. All examinations performed depend on the discretion and clinical routine of the physician.

The registry contains a basic set of variables (mandatory data) which are essential for all included patients. Another set of variables (facultative data) can be requested from the participating sites but is left to their discretion. The platform is capable for substudies (i.e., addition of further variables) in selected centres to facilitate research collaboration between institutions.

### Sites

This registry will be maintained in approximately 30 sites (hospitals and office-based) with expertise in ILD.

The number of patients per site is not limited. The study will likely be representative for expert centres in Germany.

### Patients

Patients are eligible for documentation, if they meet all of the following criteria:

- Fibrotic ILD*, which include all ILD groups including those with IIPs, CTD-ILD, cHP, asbestosis, sarcoidosis, etc.
- Age ≥ 18 years
- Interstitial lung disease on HRCT > 10% of lung parenchyma
- On active anti-inflammatory, immunomodulatory, and or anti-fibrotic therapy
- DLco ≤ 80% predicted
- Written informed consent

*Criteria of progression are not mandatory at the time of inclusion.

Patients are not eligible for documentation, if they meet one or both of the following criteria:

- Diagnosis of IPF
- Participation in a controlled trial investigating ILD, if blinded or with investigational drug

### Visit schedule and assessments

Patients should be followed up every six months – at least once per year. At this visit, relevant information since the last documentation is collected.

Documentation of data from visits can be performed at the clinic or remotely (e.g., via telephone or video chat), however, such data must be part of the patient charts (exception: data from patient-related outcomes questionnaires, if applicable). For the data collection, the following types of forms are available: inclusion, follow-up, medication, safety reporting.

Owing to the observational study type, no additional or specific visits, tests or assessments are required for the purposes of this registry.

Table 2 lists all tests and assessments recordable at the various time points, if performed on a routine basis.

**Table 2:** Assessments

Parameters (if appropriate/if performed)	Inclusion documentation	Follow up documentations every 6 months
Date of documentation	By EDC	By EDC
Date of Informed Consent	X	
Date of visit / contact with patient	X	X
Eligibility criteria	X	
Demographics	X	
Physical examination	X	X
ILD characterization Baseline information (diagnostic procedures) Symptoms Etiology	X	X
Diagnostic procedures HRCT (details t.b.d.) Other	X	X
Lung function FVC DLco FEV1, other	X	X
Exercise capacity 6-min walk distance	X	X
Concomitant diseases	X	X
Treatment History Current medication for ILD (immunosuppression, antifibrotic) Concomitant medication (anticoagulation etc.) O2 Non-pharmacological management	X	X
QoL 0–100 VAS Other instruments in substudies	X	X
Clinical events Exacerbations Hospitalization due to ILD Cardiovascular events Deaths		X
Laboratory assessments, including CRP LDH Differential blood cell count (lymphocytes, neutrophils, eosinophils, monocytes), BAL differential cell count (as available)	X	X
Safety Reporting for nintedanib Adverse Drug Reactions Fatal Adverse Events Pregnancy	X	X
Survival status		X

For exacerbations, the updated classification of exacerbations as issued by the International Working Group Report is used in a modified version [15].

Medications will be documented in detail in terms of drug name and daily dose. Relevant concomitant medications will be documented with generic drug name (INN) or trade name, dosage and with start and stop dates. ILD treatment may be interrupted or prematurely discontinued. In such cases the patient can remain in the registry.

Information on non-pharmacological therapy will be collected in categorial terms (e.g., physiotherapy).

Quality of life on a generic 0–100 numerical scale will be assessed. Other instruments/ questionnaires may be added during the course of the registry.

Patients should be followed up as long as possible (until termination of the registry or death). Patients can stay in the registry irrespective of therapy decisions (if drug therapy is switched, escalated or completely withdrawn), and also after lung transplantation.

Every patient has the right to withdraw participation at any time without giving any reason. Patient will be informed about this right in the Informed Consent form. Data collected up to the time of withdrawal will be used in the analysis and included in the clinical registry report.

Reasons for discontinuation of patients in the registry will be reported as on a follow-up documentation form (end-of-observation visit, EOO). Reasons include lost to follow-up, physician decision, patient decision (e.g. informed consent withdrawn) or administrative reasons. In case of lost-to-follow up, efforts should be made to contact the patient (by phone or other channels) in order to collect missing information.

Regardless of the reason, all data available for the subject up to the time of discontinuation should be recorded on the appropriate eCRF.

## Safety reporting

Due to sponsor obligations, serious and non-serious adverse drug reactions (ADR), fatal adverse events (AE), and pregnancies in female patients (and in partners of male patients) in all patients receiving nintedanib must be reported by investigators to Boehringer Ingelheim. The described events are to be reported by the electronic data entry form on the website of the INSIGHTS-ILD registry. In case the electronic system should not be accessible, the investigator may alternatively use paper sheets which should be sent via fax to Boehringer Ingelheim.

Serious ADR and fatal AE must be reported within 1 business day after knowledge. Non-serious ADR (e.g., all ADR that do not meet the definition of serious) and pregnancies should be reported within 7 business day after knowledge.

## Data collection and quality control

The Sponsor is responsible for implementing and maintaining a quality management system with written development procedures and functional area standard operating procedures (SOP) to ensure that studies are conducted and data are generated, documented, and reported in compliance with the protocol, accepted standards of Good Epidemiological Practice (GEP) [16], and all applicable laws, rules and regulations relating to the conduct of the registry.

Before the registry starts at the sites, all investigators will be sufficiently trained on the background and objectives of the study and ethical as well as regulatory obligations.

Data will be collected by the centers online, using a secure connection. No paper pencil CRFs will be used. The electronic data capture system (EDC) with electronic data collection forms (eCRFs) and other features (e.g., repository of registry documents) will be provided by the sponsor. No software applications need to be installed by the sites. The web-based EDC application is password protected. User account requests are authorized by GWT. A manual with instructions how to use the EDC will be made available to sites.

The data will be entered by the investigator or registry site personnel via a secure internet connection into the registry database. Only these persons can record or change data relating to patients in their center on the CRFs.

Detailed information on checks for completeness, accuracy, plausibility and validity are given in the Data Management Plan (DMP). The same plan will specify measures for handling of missing data and permissible clarifications. The DMP is available upon request.

Upon entry, the data is automatically checked for completeness and plausibility.

As part of the statistical processing, the data is checked again and queries are sent to the Centre if necessary. If needed, requests will be sent to the sites to correct incorrect inaccurate data or complete missing data.

In about 10% of the centers, an audit will be carried out to check the correct data entry while respecting data protection (monitoring with comparison of the registry data with original patient data).

## Statistical considerations

### Sample size

As the present registry has mainly descriptive aims, there is no formal power calculation needed to determine the target sample size [17]. Rather, the sample size is determined by feasibility aspects.

From a drug safety angle, an enrolled number of 900 patients according to the “rule of three” will allow for the detection of ADR occurring at an incidence of 1 in 300 person-years [18, 19]. If the median observation duration is 2.5 years, an ADR occurring with an incidence of 1 in 750 person-years will be detected.

### Descriptive analysis and statistical testing

Data will be analyzed in 6-month intervals in order to keep sites appraised of the progress of the registry and to review data quality (rate of missing values, data distribution, outliers).

Concomitant medication will be coded using the WHO Drug Dictionary and tabulated by ATC term. Medical history, concomitant diseases and ADR will be coded by MedDRA in its current version [20].

All background variables and outcome parameters will be analyzed descriptively at baseline and each subsequent follow-up assessment with appropriate statistical methods: categorical variables by frequency tables (absolute and relative frequencies) and continuous variables by summary statistics (i.e., mean, standard deviation, minimum, median, quartiles and maximum). Continuous variables will also be described by absolute value and as change from baseline per analysis time point, if applicable. The primary endpoint as well as secondary endpoints will be analyzed in regular intervals.

Analyses will be performed for the total study population (overall analysis) and separately by indication or relevant subgroups (e.g., PPF groups, age, sex, pretreated and newly treated patients), if patient numbers are sufficient. Pretreated patients are here defined as patients already on treatment when enrolled in the study, whereas newly treated patients will be patients newly starting ILD treatment.

Survival outcome and clinical events such as exacerbations will be analyzed by Kaplan Meier estimates. Association of mortality and clinical events with parameters of interest will be analyzed by Cox proportional hazard model with time-invariant and time-dependent covariates. The change in lung function parameters (FVC, DLCO), 6MWD, QoL and days of hospitalization will be modeled by generalized linear mixed models to account for the longitudinal data. Potential predictor variables will be included into the generalized linear mixed models to determine correlates for the analyzed outcome variables. The change in treatment strategy and drug utilization (dose and dosing schedule, duration of treatment (persistence), switches between treatments, phases without drug treatment, and reasons for drug discontinuation) will be descriptively reported. Exploratory analyses will be performed in order to determine associations of biomarkers with the defined outcome variables.

Comparison of patients treated with and without antifibrotic therapy in terms of mortality, pulmonary function test results and other outcomes will be performed on group level and not on single drug level (nintedanib, pirfenidone) as described in the following. The entire observation period will be considered for each patient in the registry in order to compare outcomes of interest. The observation time will start for each patient at registry enrolment in order to avoid immortal time bias (= “waiting time bias” bias due to exposure misclassification). Patients may start and stop new treatments such as antifibrotic therapy during follow-up. All available treatment episodes reported in the registry will be used in order to analyze the association between treatments and outcomes. At least two treatment periods will be assigned to patients who started antifibrotic therapy during follow-up; namely, a treatment period without antifibrotic therapy after enrolment, and another with antifibrotic therapy after treatment initiation. The episode before the start of antifibrotic therapy will be included in the mortality and other outcome analyses of the “no antifibrotics” group to account for immortal time bias. The analyses will be performed by generalized mixed models including time-dependent covariates. All analyses are planned on the group level (antifibrotic therapy yes / no), the exposition time to any antifibrotic therapy is used for analyses. Information on the specific drug is descriptively provided (percentage of patients on the drugs at BL, 6, 12, 18, 24 and 30 months). Detailed information (on product, dose and treatment dates) on previous (and current) therapy for pulmonary fibrosis will be collected.

Comparison of the two treatment groups in terms of lung function data and 6MWD test results will be analyzed by propensity score weighted linear mixed models to account for the possibility of more than one treatment episode for a single patient (additional cluster variable), and to account for the longitudinal study design, which was based on observed values. Intention to treat analyses will be additionally performed if it will be appropriate.

Further statistical methods planned include propensity score technique, multiple imputations, competing risks analyses, and latent class analysis to determine cluster of patients.

All statistical details including calculated variables and proposed format and content of tables will be detailed in the SAP, which will be finalized before study database lock.

## Discussion

The study has been registered in the NIS registry of the BfArM under NIS7562, and in the DRKS registry under DRKS00027389. Study results will be reported after termination of the project on the BfArM website as Clinical Study Report. The results of this registry are intended to be published in a series of articles in peer-reviewed journals and as abstracts/presentations at medical congresses under the oversight of the sponsor. Current guidelines and recommendation on good publication practice will be followed (e.g. STROBE [21]).

The study was initiated by investigators (see authors), the legal sponsor is the GWT-TUD in Dresden. The study entered the field phase in December 2021, and has included 360 patients to date (11 January 2023). The field (documentation) phase of the study is planned until December 2024. All study materials are available in English.

## Data Availability

Data sharing is not applicable to this article as datasets are being generated but have not been analyzed during the current study.

## Abbreviations

6-MWD: 6-Minute Walk Distance
AE: Adverse Event
ADR: Adverse Drug Reaction
BAL: Bronchoalveolar lavage
BfArM: Bundesinstitut für Arzneimittel und Medizinprodukte (BfA
BMI: Body Mass Index
CI: Confidence Interval
cHP: Chronic hypersensitivity pneumonitis
CRP: C-reactive protein
DL_CO_: Diffusion capacity for carbon monoxide
DRKS: Deutsches Register Klinischer Studien
eCRF: Electronic Case Report Form
EDC: Electronic Data Capture
ESC: European Society for Cardiology
ERS: European Respiratory Society
FVC: Forced vital capacity
GDPR: General Data Protection Regulation
GEP: Good Epidemiological Practice
GWT: Gesellschaft für Wissensschafts- und Technologietransfer
HRCT: High resolution chest computer tomography
ICF: Informed Consent Form
ICMJE: International Committee of Medical Journal Editors
IEC: Institutional Ethics Committee
EOO: End of observation
IIP: Idiopathic interstitial pneumonia
ILD: Interstitial lung disease
INN: International Nonproprietary Name
IPF: Idiopathic pulmonary fibrosis
LTOT: Long-term oxygen therapy
LTx: Lung transplantation
MMF: Mycophenolate mofetil
NHLBI: National Heart, Lung, and Blood Institute
NIS: Non-interventional Study
NYHA: New York Heart Association
PRO: Patient related outcomes
SAE: Serious Adverse Event
SAP: Statistical Analysis Plan
SIC: Subject Identification Code
SmPC: Summary of Product Characteristics
SOP: Standard Operating Procedures
STROBE: Strengthening the Reporting of Observational Studies in Epidemiology
TLC: Total lung capacity
Pa_O2_: Oxygen pressure
PF-ILD: Progressive fibrosing interstitial lung disease
PPF: Progressive pulmonary fibrosis
QoL: Quality of life
UIP: Usual interstitial pneumonia
VO_2max_: Maximal oxygen consumption
WHO: World Health Organization
